# Gene-Agnostic Therapeutic Strategies for Inherited Retinal Diseases: Neuroprotection and Immunomodulation

**DOI:** 10.3390/genes17040392

**Published:** 2026-03-30

**Authors:** Lucas W. Rowe, S. Patricia Becerra, Robert E. MacLaren, Robert L. Avery, Charles C. Wykoff, Allen C. Ho, Carl D. Regillo, Dean Eliott, Andrew Osborne, Katie M. Binley, Thomas A. Ciulla

**Affiliations:** 1 Department of Ophthalmology, Glick Eye Institute, Indiana University School of Medicine, Indianapolis, IN 46202, USA; 2 Laboratory of Retinal Cell and Molecular Biology, Section of Protein Structure and Function, National Eye Institute, National Institutes of Health, Bethesda, MD 20892, USA; becerras@nei.nih.gov; 3 Nuffield Laboratory of Ophthalmology, Department of Clinical Neurosciences, University of Oxford, Oxford OX3 9DU, UK; 4 Oxford University Hospitals NHS Foundation Trust, Oxford OX3 9DU, UK; 5 California Retina Consultants, Santa Barbara, CA 93103, USA; 6 Retina Consultants of Texas, Bellaire, TX 77401, USA; 7 Wills Eye Hospital, Mid Atlantic Retina, Thomas Jefferson University, Philadelphia, PA 19107, USA; 8 Massachusetts Eye and Ear Infirmary, Harvard Medical School, Boston, MA 02115, USA; 9 Ikarovec Ltd., Norwich NR4 7GJ, UK; aosborne@ikarovec.com (A.O.); kbinley@ikarovec.com (K.M.B.); 10 Retina Service, Midwest Eye Institute, Indianapolis, IN 46290, USA

**Keywords:** inherited retinal disease, gene therapy, neuroprotection, immunomodulation, gene agnostic

## Abstract

**Background/Objectives**: Inherited retinal diseases (IRDs) represent a genetically heterogeneous group of disorders caused by mutations in over 280 genes with more than 3100 identified variants. While gene-specific replacement therapies have achieved landmark success with voretigene neparvovec (Luxturna) for biallelic *RPE65*-associated retinal dystrophy, developing individual therapies for each genetic subtype remains impractical. This review examines gene-agnostic therapeutic approaches utilizing neuroprotection and immunomodulation that target common pathophysiological mechanisms shared across multiple IRD genotypes. **Methods**: We reviewed the literature on neuroprotective and immunomodulatory gene therapy strategies for IRDs, focusing on neurotrophic factors and complement system modulation. **Results**: Neuroprotective approaches delivering neurotrophic factors—including pigment epithelium-derived factor (PEDF), ciliary neurotrophic factor (CNTF), rod-derived cone viability factor (RdCVF), brain-derived neurotrophic factor (BDNF), fibroblast growth factors (FGFs), glial cell line-derived neurotrophic factor (GDNF), and proinsulin—have demonstrated photoreceptor preservation across multiple preclinical IRD models regardless of the underlying genetic mutation. The recent FDA approval of CNTF cell-based gene therapy (Encelto) for macular telangiectasia type 2 validates this therapeutic paradigm. Complement system inhibition represents another gene-agnostic strategy, with intravitreal complement inhibitors approved for geographic atrophy secondary to age-related macular degeneration and gene therapy approaches targeting C3, C5, or delivering soluble complement regulators under investigation for IRDs. Combination strategies simultaneously addressing multiple pathogenic pathways may offer synergistic benefits. **Conclusions**: Gene-agnostic approaches targeting neuroprotection and immunomodulation offer a therapeutic paradigm capable of benefiting patients across the spectrum of IRD genotypes, potentially transforming treatment for conditions where mutation-specific therapies remain unavailable.

## 1. Introduction

Inherited retinal diseases (IRDs) represent a leading cause of irreversible blindness in both children and the working-age population, collectively affecting millions worldwide with profound impact on patients and society [1]. Clinicians regularly encounter patients and families facing the challenge of progressive vision loss with no approved treatment options. These disorders are characterized by progressive degeneration of photoreceptors or the retinal pigment epithelium (RPE), leading to visual acuity loss, visual field constriction, or both, ultimately progressing to legal blindness in most affected individuals. Although the landmark approval in 2017 of voretigene neparvovec-rzyl (Luxturna, Spark Therapeutics, Philadelphia, PA, USA) for confirmed biallelic *RPE65*-mediated retinal dystrophy validated the feasibility of adeno-associated viral (AAV)-based gene replacement therapy [2], this success underscores the limitations of gene-specific approaches. Notably, biallelic *RPE65*-mediated IRD accounts for approximately 1% of all IRDs, highlighting that even successful gene-specific therapies address only a small fraction of the affected patient population [3].

A major barrier is the genetic heterogeneity underlying IRDs. More than 3100 pathogenic alleles across over 280 genes have been identified as causal for IRDs [4,5,6]. While it is becoming increasingly possible to develop gene therapies for rare diseases, including efforts to streamline and standardize manufacturing methods, significant hurdles remain in identifying patient-relevant genes, designing and developing gene therapy vectors, conducting preclinical evaluation, and advancing through clinical trials [7]. This presents a task that is both scientifically and economically challenging, particularly given the rarity of individual mutations. Furthermore, many patients remain genetically undiagnosed, effectively excluding them from gene-targeted treatments. Even patients with clear phenotypic features of IRD cannot access Luxturna or enroll in gene replacement trials if their causative mutation has not been identified. Additionally, gene augmentation in genetically confirmed cases may be limited by factors such as large gene size, which poses challenges for genetic delivery, dominant-negative mutations, or advanced photoreceptor loss that can preclude meaningful functional recovery [8]. Furthermore, for many confirmed genetic diagnoses, no gene replacement therapy exists or is under active development, leaving supportive care as the only available option.

**Figure 1:**
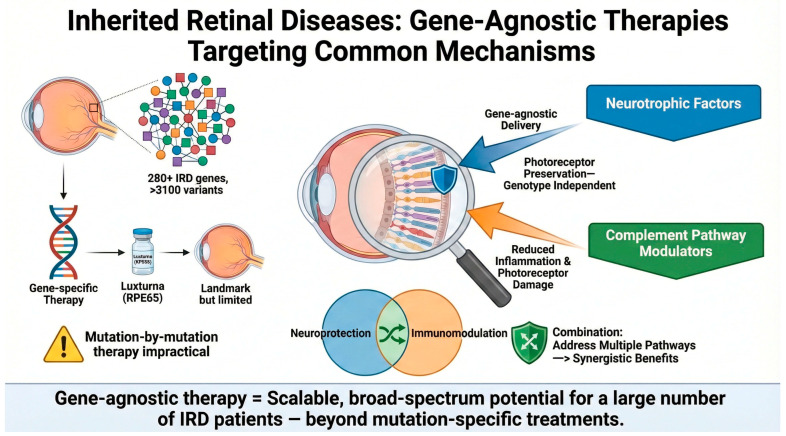
Overview of the rationale supporting gene-agnostic therapeutic strategies for IRDs.

These constraints have catalyzed growing interest in gene-agnostic therapeutic strategies capable of providing benefit irrespective of genotype [9,10]. While the primary driver of IRD pathology is the underlying genetic mutation, this triggers a cascade of downstream consequences including oxidative stress, metabolic dysfunction, complement dysregulation, and neuroinflammation that ultimately converge on photoreceptor cell death. Gene-agnostic approaches target these common downstream pathological pathways rather than the primary genetic defect (Figure 1). Such approaches aim to preserve or restore vision by targeting common mechanisms of degeneration including neuroprotection, inflammation, oxidative stress, and metabolic dysfunction. While these strategies bypass the need for mutation-specific interventions, their efficacy may vary depending on the underlying disease mechanism, stage of progression, and remaining photoreceptor reserve. From an economic perspective, a gene-agnostic therapeutic approach would be more scalable and cost-effective than individualized gene-specific gene therapies [11]. It is important to note that these gene-agnostic strategies are disease-modifying rather than curative; they do not correct the underlying genetic defect but instead aim to delay or halt disease progression and prevent photoreceptor degeneration. As such, they may serve as standalone therapies for patients without access to gene-specific treatments, or as adjunctive therapies to complement gene replacement approaches.

**Figure 2:**
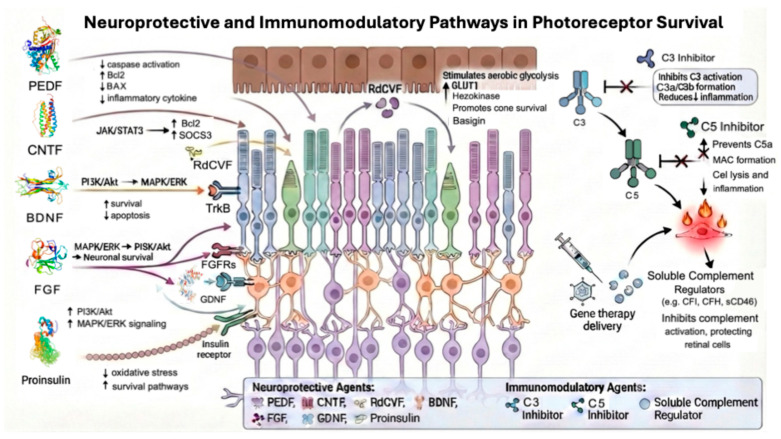
Schematic overview of the major neuroprotective and immunomodulatory therapeutic pathways for IRD.

This review focuses on preservation-oriented strategies aimed at slowing or halting photoreceptor degeneration, focusing on neurotrophic factor delivery and complement modulation strategies (Table 1 and Figure 2). Alternative gene-agnostic strategies include modifier gene therapy approaches such as OCU400 (AAV-h*NR2E3*, Ocugen, Malvern, PA, USA), which delivers the *NR2E3* transcription factor via AAV5 to restore photoreceptor homeostasis; this approach is mechanistically distinct from the neurotrophic factor delivery and complement modulation strategies that are the focus of our review [12]. Similarly, optogenetic approaches that target patients with end-stage retinal disease with profound vision loss, confer light sensitivity to remaining inner retinal cells, and represent a complementary gene-agnostic paradigm that is reviewed elsewhere.

## 2. Neuroprotective Approaches

Neuroprotective interventions seek to preserve existing photoreceptors and RPE, thereby delaying or preventing irreversible visual loss. These therapies act broadly, independent of specific genetic mutations, by stabilizing cellular metabolism, reducing cell death, and modulating trophic signaling [13,14].

### 2.1. Growth Factor-Based Therapies

#### 2.1.1. Pigment Epithelium-Derived Factor (PEDF)

PEDF is a 50 kDa glycoprotein secreted by the RPE with neurotrophic, anti-angiogenic, and anti-inflammatory properties [15]. PEDF levels in the eye decline with retinal degeneration [16], age-related macular degeneration (AMD) [17], and age [18]. Furthermore, loss of PEDF may cause age-related changes in the retina and RPE [19]. Its neuroprotective mechanisms include promotion of photoreceptor survival, reduction in oxidative stress, and regulation of apoptotic pathways [20,21]. Its neuroprotective actions are mediated largely through its receptor PEDF-R (*PNPLA2*) on photoreceptor inner segments [22,23,24]. Mechanistically, PEDF preserves calcium homeostasis via activation of the plasma membrane calcium pump (PMCA) and preventing calcium-induced calpain/BAX/apoptosis-inducing factor (AIF)-mediated cell death while also inhibiting AIF nuclear translocation and increasing Bcl-2 expression [25,26]. Additionally, PEDF promotes photoreceptor differentiation, rhodopsin polarization, and neurite outgrowth [27]. These pleiotropic mechanisms position PEDF as a broad neuroprotective agent targeting multiple IRD-associated pathways [24].

PEDF has demonstrated robust neuroprotective efficacy in multiple rodent models of IRD [28,29]. In *rd10* mice, which carry a mutation in the *PDE6B* gene causing progressive rod photoreceptor death, PEDF protein levels in the RPE decline with age, coinciding with photoreceptor degeneration [30]. Furthermore, PEDF deficiency in *SERPINF1* null mice crossed with *rd10* mice resulted in accelerated retinal degeneration compared to *rd10* mice alone, implying PEDF’s protective role in IRD and demonstrating that PEDF deficiency increases susceptibility to retinal degeneration [31].

Multiple delivery strategies for pigment epithelium–derived factor (PEDF), including topical eye drops and direct ocular injection, have demonstrated sustained intraocular bioactivity with retinal exposure at physiologically relevant concentrations [32,33]. PEDF-derived peptides, particularly 17-mer fragments encompassing the neurotrophic domain that interacts with the PEDF receptor (PEDF-R), have likewise been formulated as eye drops and shown to penetrate ocular tissues and reach the retina [34]. Intravitreal administration of PEDF or these 17-mer peptides conferred significant protection of cone photoreceptors from LED-induced phototoxicity in rodent models [29,35]. In *RhoP23H*/+ mice and other models of retinitis pigmentosa (RP), vector-mediated ocular gene delivery of PEDF prevented photoreceptor cell death, inhibited apoptosis-inducing factor (AIF) nuclear translocation, and preserved retinal structure over extended periods [25]. Consistently, topical delivery of the 17-mer peptide partially prevented photoreceptor loss, reduced apoptosis, and increased rod and cone gene expression in microphthalmia (*Mitf)* mutant mice with RPE-associated retinal degeneration [36]. Administration of recombinant PEDF, PEDF-derived peptides, or the neurotrophic variant H105A increased outer nuclear layer (ONL) thickness and diminished photoreceptor degeneration in *rd10* retinal explants, *rd10* mice in vivo, and human retinal organoids exposed to cigarette smoke extract–mediated stress [30,34]. Notably, topical delivery of H105A achieved retinal penetration, increased rhodopsin and opsin levels, reduced cell death markers (BAX/Bcl2 ratio), preserved photoreceptor survival for up to six months, and improved retinal function as assessed by electroretinography in both *rd10* and *RhoP23H*/+ mice [34]. In human retinal organoids subjected to oxidative stress, H105A similarly reduced photoreceptor cell death [34]. Collectively, these findings support PEDF as a receptor-dependent neuroprotective factor across diverse genetic models of inherited retinal degenerations.

Gene therapy vector mediated delivery of PEDF has also shown positive results in preclinical models. For example, intravitreal delivery of an adenovirus-based vector expressing PEDF in Lewis rats significantly protected photoreceptors from light-induced apoptotic cell death, preserving photoreceptor density and improving ERG function compared to control eyes [37]. Subretinal injection of a third-generation lentiviral vector based on simian immunodeficiency virus (SIV-hPEDF) has achieved safe and stable transgene expression of PEDF maintained for over five years in nonhuman primates and has also shown a significant delay of retinal degeneration in rodent RP models [38,39,40,41].

Despite promising preclinical results, clinical translation remains limited for pathology in the posterior segment of the eye due to challenges in dosing and durability. For instance, the inverse scaling of vitreous volume to ocular surface area between mouse and human eye raises significant challenges in achieving therapeutic levels following topical application. Only a single phase 1 clinical trial has been conducted to date, evaluating PEDF for choroidal neovascularization [42]. The study found that adenoviral vector-delivered PEDF (ADGVPEDF.11D) in patients with neovascular AMD demonstrated safety with no serious adverse events and showed dose-dependent antiangiogenic activity [43]. A phase 1/2a investigator-initiated clinical trial (jRCT2073180024) to evaluate the safety and efficacy in patients with RP is ongoing; however, efficacy and safety data are not available at the time of publication [44]. The relatively short half-life of recombinant PEDF protein necessitates frequent administrations, which is impractical for chronic retinal diseases [45,46,47]. While frequent intravitreal administration (e.g., every 3 months for some RNA therapies) may be considered a disadvantage compared to a one-time gene therapy approach, this treatment burden is comparable to or less than that required for approved intravitreal therapies for acquired macular diseases, which may require monthly injections. While gene therapy offers sustained expression, questions remain regarding optimal vector design, route of administration, immune responses to vector components, and long-term safety [48,49]. Future optimization of delivery routes, vectors and sustained release systems will be essential to realize PEDF’s therapeutic potential in human IRDs.

#### 2.1.2. Ciliary Neurotrophic Factor (CNTF)

CNTF promotes photoreceptor survival through the JAK/STAT pathway activation and modulation of Müller glial responses [50,51,52]. In multiple rodent and canine models, CNTF slowed photoreceptor loss and maintained ONL integrity by direct neuroprotection, modulation of glial responses, and metabolic support, with efficacy demonstrated across multiple delivery platforms [53,54,55]. Translation of these promising preclinical neuroprotective results in human retinal degenerative diseases has proved to vary by disease and outcome measure. Several factors contribute to this variable translation, including: (1) disease stage at intervention, as neuroprotective agents require the target cells to be viable to exert their effects; (2) baseline photoreceptor reserve, which determines the therapeutic ceiling; (3) degree of retinal remodeling, which can disrupt the cellular architecture and synaptic connectivity necessary for functional recovery; and (4) species differences in retinal anatomy and physiology that may limit the predictive value of preclinical models.

Revakinagene taroretcel-lwey (Encelto, Neurotech Pharmaceuticals, Cumberland, RI, USA) was approved in March 2025 by the United States Food and Drug Administration (FDA) as the first therapy for the treatment of adults with idiopathic macular telangiectasia type 2 (MacTel) [56]. Revakinagene taroretcel-lwey is an allogeneic encapsulated cell-based gene therapy providing sustained delivery of recombinant CNTF (rhCNTF) to promote the survival and maintenance of photoreceptors. The genetically engineered RPE cells are contained in an intravitreal implant which is surgically implanted to the sclera at the pars plana, leading to continuous production and release of CNTF into the vitreous cavity. FDA approval was based on the results of two phase 3 trials which demonstrated that revakinagene taroretcel-lwey significantly slowed the loss of macular photoreceptors, as measured with optical coherence tomography (OCT), in MacTel patients over 24 months. Revakinagene taroretcel-lwey significantly slowed the rate of ellipsoid zone (EZ) area loss compared to sham in both NTMT-03-A (NCT03316300; 0.075 vs. 0.166 mm^2^ over 24 months; difference: −0.091 mm^2^, *p* < 0.0001) and NTMT-03-B (NCT03319849; 0.111 vs. 0.160 mm^2^; difference: −0.049 mm^2^, *p* = 0.0186) [57]. In addition, the studies demonstrated functional benefit in patients who received the CNTF implant, as reading speed loss was substantially reduced in the treatment groups compared to the control groups.

In RP, multicenter, sham-controlled trials (NCT01530659, NCT00447980, NCT00447993) found that CNTF delivered via intraocular encapsulated cell implants was safe and achieved long-term intraocular protein release [58,59]. However, these studies did not show significant benefit in primary visual function endpoints (visual acuity or field sensitivity). In fact, long-term follow-up showed greater visual field loss from baseline than sham eyes, which was reversible upon removing the implant [59]. Some secondary outcomes, such as increased retinal thickness and stabilization of vision in subgroups, were observed, but overall, efficacy was less robust than in animal models [58]. This discrepancy between preclinical and clinical outcomes may be attributed to several factors: (1) Species differences: rodent models have shorter disease time courses and different central retinal architecture compared to humans; (2) Timing of intervention: in preclinical studies therapeutic intervention is often initiated before or shortly after disease onset, whereas human patients typically present with established disease; (3) Disease chronicity: the prolonged duration of human IRDs allows for extensive retinal remodeling and circuit disruption that may not be captured in short-term animal studies; and (4) Outcome measures: functional endpoints in humans may be less sensitive to detecting neuroprotective effects than the histological endpoints commonly used in preclinical studies. The divergent outcomes observed across different retinal diseases likely reflect, in part, differences in disease stage at the time of intervention. Because CNTF supports surviving photoreceptors rather than restoring lost cells, its efficacy is inherently stage-dependent. In the RP trials, enrolled patients often had advanced disease with significant photoreceptor loss at baseline, potentially limiting the therapeutic window. In contrast, MacTel patients typically retain better baseline photoreceptor structure in the macula. These observations underscore the importance of early intervention and appropriate patient selection in future neuroprotective trials.

For geographic atrophy (GA) secondary to AMD, a phase 2 trial found that high-dose CNTF stabilized visual acuity and increased retinal thickness compared to sham, especially in patients with better baseline vision [60]. In the GA trial, the stabilization effects were observed through 12 months, with the most pronounced benefits seen in patients with better baseline visual acuity (20/63 or better), suggesting that earlier intervention when more photoreceptors remain viable may be associated with better outcomes. Long-term durability beyond 12 months has not been reported for this indication. Further trials are being carried out for visual restoration in glaucoma (NCT04577300, NCT01408472, NCT02862938) and achromatopsia (NCT01648452) showing the potential breadth for a gene agnostic approach outside of a single disease. In the latter case, CNTF has been observed to increase photoreceptor outer segment growth, and may have the potential to augment achromatopsia gene replacement therapies [61].

#### 2.1.3. Rod-Derived Cone Viability Factor (RdCVF)

RdCVF is a thioredoxin-like protein secreted by rod photoreceptors that maintains cone viability by promoting glucose uptake through the basigin-1 (BSG1)/GLUT1 complex. RdCVF binds to BSG1 on cone photoreceptors, which interacts with GLUT1 to increase glucose entry into cones, thereby increasing the available pool of intracellular glucose to support cone metabolic demands and outer segment renewal. This mechanism is central to the secondary cone degeneration observed in IRDs, where rod loss leads to a reduction in RdCVF secretion, depriving cones of the metabolic support necessary for survival [62].

In *rd1* and *rd10* mouse models, delivery of RdCVF as a recombinant protein or via a gene therapy markedly preserved cone structure and visual function. AAV-mediated expression of RdCVF in these models resulted in delayed cone loss, improved photopic ERG responses, and sustained visual acuity [63,64,65]. These effects are largely mutation-independent, as RdCVF acts on a metabolic pathway common to all cones. However, it should be noted that the degree of cone metabolic vulnerability and responsiveness to RdCVF may vary across different IRD subtypes, particularly in conditions where primary cone dysfunction or structural abnormalities may limit the therapeutic response.

Given its gene-independent mechanism and ability to rescue cones metabolically, RdCVF is considered one of the most promising broad-spectrum therapeutic candidates for IRDs. Preclinical safety and efficacy data have supported the initiation of first-in-human studies of RdCVF for IRD [63,64,66]. SPVN06 (SparingVision, Paris, France) is a subretinal AAV-based gene therapy delivering both RdCVF and Rod derived Cone Viability Factor Long form (RdCVFL), an enzyme which protects cones against oxidative stress. The ongoing Phase I/II PRODYGY trial (NCT05748873) evaluating SPVN06 in subjects with advanced rod-cone dystrophy (RCD) due to mutations in the *RHO*, *PDE6A*, or *PDE6B* gene has demonstrated a strong safety profile up to one year after injection, with no significant intraocular inflammation or immune response [67]. Specific peer-reviewed human efficacy data remain pending.

In summary, RdCVF’s role in promoting cone glucose uptake via the BSG1/GLUT1 complex provides a direct mechanistic link between rod loss and cone degeneration, and its mutation-independent rescue of cones positions it as a leading candidate for IRD therapy, with clinical translation underway.

### 2.2. Other Neuroprotective Strategies

Additional neurotrophic and metabolic factors including brain-derived neurotrophic factor (BDNF), fibroblast growth factors (FGFs), glial-derived neurotrophic factor (GDNF), and metabolic modulators like proinsulin have shown protective effects across various retinal degeneration models. Although translation of these agents to clinical use remains preliminary, the breadth of preclinical evidence underscores the potential of multi-factor neuroprotection as a gene-agnostic therapeutic platform.

BDNF has been extensively studied for its neuroprotective and restorative effects in retinal diseases. Sustained BDNF expression in the retina delays photoreceptor cell death and preserves retinal function in models of IRD and oxidative damage, primarily through tropomyosin receptor kinase B (TrkB) receptor signaling and anti-apoptotic pathways [68,69,70]. Gene therapy approaches, such as AAV-mediated BDNF/TrkB delivery, have achieved sustained neuroprotection and long-term signaling enhancement in preclinical models, with no adverse effects on retinal structure or function [71,72]. These strategies are advancing toward clinical trials, with novel nanoparticle platforms enabling efficient and safe delivery to Müller cells and demonstrating synergistic effects when combined with metabolic modulators like oligomycin [73]. However, translation to routine clinical use is limited by challenges in maintaining therapeutic levels and receptor downregulation.

FGFs, particularly FGF2, have shown significant photoreceptor rescue in IRD models. Intravitreal FGF2 improves photoreceptor morphology and, when combined with agents like minocycline, yields additive neuroprotective effects by reducing microglial activation and enhancing cell survival [68,74]. While FGF2 and other FGFs are recognized for their neuroprotective potential, clinical translation is still preliminary, with most evidence derived from preclinical studies.

GDNF and related neurotrophic factors play interdependent roles in retinal neuroprotection, modulating neuronal survival, differentiation, and glial responses. GDNF has demonstrated efficacy in models of diabetic retinopathy and IRD, often acting synergistically with other trophic factors [70,75]. GDNF, alone or in combination with BDNF, has been safely delivered via sustained-release microspheres in animal models, supporting RGC survival and migration of RPE cells without toxicity or apoptosis [76]. These delivery systems are being developed for long-term intravitreal administration, with the potential for personalized dosing in future clinical applications.

Metabolic modulators such as proinsulin exert neuroprotective effects by attenuating oxidative stress, enhancing mitochondrial integrity, and supporting cell survival. Proinsulin is the single-chain precursor molecule of insulin, which is distinctive from insulin-like growth factor 1 (IGF-1), a peptide hormone primarily involved in growth regulation. Proinsulin activates the insulin receptor-A pathway in the retina, preserving synaptic connectivity and prolonging visual function in RP models without systemic metabolic effects [77]. These agents reduce neuroinflammation and promote metabolic efficiency, directly countering the metabolic deficits seen in degenerating retinas. These approaches are highlighted as promising gene-agnostic strategies, but human clinical data remain limited [78].

While multi-factor neuroprotective agents have demonstrated strong preclinical efficacy and safety, most are still progressing through proof-of-concept and safety studies. The major challenge is translating robust animal model results into meaningful clinical outcomes, with ongoing trials focused on refining delivery, patient selection, and endpoints [78,79].

## 3. Regulation of the Complement System

### 3.1. Complement Dysregulation in IRDs

The complement cascade, traditionally viewed as a defense mechanism, is increasingly recognized as a contributor to chronic retinal inflammation and photoreceptor loss in both acquired and inherited retinal degenerative diseases. Whether complement dysregulation represents a primary driver of degeneration or a secondary response to ongoing cell death remains an area of active investigation. Evidence suggests that complement activation may begin as a secondary response to photoreceptor stress and death but subsequently becomes a pathogenic amplifier that accelerates degeneration through a feed-forward mechanism. This distinction has important therapeutic implications: if complement activation is a secondary response, then its inhibition would be expected to slow rather than halt disease progression, consistent with the disease-modifying rather than curative nature of this approach. Evidence of complement dysregulation has been documented in Stargardt disease, RP, and Leber congenital amaurosis, where upregulation of C3 and C5 components correlates with findings in AMD [80,81,82,83,84,85]. However, important differences exist between these conditions. AMD is primarily an age-related, multifactorial disease with strong environmental contributions, whereas IRDs result from single-gene mutations with earlier onset and distinct temporal dynamics of degeneration. Nevertheless, the convergence on complement pathway dysregulation in both conditions suggests this may represent a common final pathway amenable to therapeutic intervention. Overactivation of complement can trigger microglial recruitment, cytokine release, and formation of the membrane attack complex (MAC), which exacerbates cell death and accelerates retinal degeneration.

In Stargardt disease, both human donor eyes and mouse models show increased deposition of C3 fragments and MAC on the RPE, with reduced levels of complement regulatory proteins such as factor H. This dysregulation leads to chronic inflammation and accelerates photoreceptor degeneration. Gene therapy to increase complement regulation in the RPE (e.g., AAV-CRRY, AAV-*RORA*) reduces C3 activation, MAC deposition, and slows photoreceptor loss in mouse models, directly implicating complement activation in disease pathogenesis [80,81,86].

In RP, upregulation of complement components coincides with photoreceptor degeneration and microglial activation. C3 and its receptor CR3 on microglia mediate both protective clearance of apoptotic photoreceptors and, when dysregulated, may contribute to neurotoxicity and chronic inflammation [82]. Overactivation of complement can trigger microglial recruitment, cytokine release, and MAC deposition, potentially accelerating death of stressed but potentially recoverable photoreceptors. Similar complement dysregulation is observed in Leber congenital amaurosis and other IRDs.

It is important to recognize that complement plays an important physiological role in the retina beyond pathological inflammation. Complement components contribute to synaptic pruning during development, clearance of cellular debris and apoptotic cells, and immune surveillance against pathogens. Chronic complement inhibition may therefore have unintended consequences, including impaired debris clearance that could lead to accumulation of toxic metabolites, reduced immune surveillance increasing susceptibility to infection, and potential disruption of retinal homeostasis. The goal of a complement-targeted therapy is not to completely suppress complement activity but rather to restore it to a normal homeostatic level. This is reflected in the design of current therapies, which aim to dampen pathological overactivation while preserving baseline physiological function. Long-term safety monitoring in ongoing and future trials will be essential to evaluate these potential risks.

In summary, complement overactivation is a common pathogenic mechanism in AMD and IRDs that drives chronic inflammation and photoreceptor loss. However, the complement pathway also plays a critical role in retinal health. Therefore, therapies that restore complement activity to normal homeostatic levels—rather than fully suppressing it—offer a promising, gene-agnostic approach to reducing retinal inflammation and preserving vision in these disorders.

### 3.2. Complement Modulation as a Therapeutic Strategy

Current anti-complement strategies for IRDs include intravitreal pharmacologic agents targeting central complement components (C3 and C5 inhibitors) and gene therapy approaches designed to provide sustained intraocular complement regulation. Recent FDA approvals of the C3 inhibitor pegcetacoplan (Syfovre, Apellis Pharmaceuticals, Waltham, MA, USA) and the C5 inhibitor avacincaptad pegol (Izervay, Astellas Pharma, Tokyo, Japan) have advanced the management of GA secondary to dry AMD. These agents have not yet been approved or systematically studied in IRDs but reducing pathological complement activation offers a rational, inflammation-modulating approach to slow disease progression in IRDs [87].

There is strong and growing preclinical and clinical evidence that dampening complement activity within the retina can preserve retinal structure, reduce inflammation, and delay secondary degeneration in IRDs [87,88,89]. In mouse models of retinal degeneration, pharmacologic or genetic attenuation of C3 and C5 reduces complement activation, microglial recruitment, and formation of MAC, leading to decreased photoreceptor loss and preservation of retinal structure [90]. For example, downregulation of both classical and alternative pathway C3 and C5 convertases was required to reduce progressive rod and cone degeneration in models of retinal atrophy, highlighting the importance of targeting both arms of the cascade for maximal protection [90]. Building upon its approval in AMD, avacincaptad pegol had been evaluated in a phase 2b clinical trial for autosomal recessive Stargardt disease 1 (STGD1) (NCT03364153), with endpoints including safety, atrophy progression, and visual function, but clinical results are not yet available [91]. Safety data from AMD trials indicate a favorable profile, with most adverse events related to the injection procedure and an increased risk of macular neovascularization. Hemorrhagic occlusive retinal vasculitis (HORV) is a rare but serious adverse event reported with intravitreal complement inhibitors. While the incidence appears low, the potentially devastating visual consequences underscore the importance of careful patient selection, post-injection monitoring, and prompt recognition and management of any signs of retinal vasculitis.

Gene therapy approaches using AAV vectors to deliver complement regulatory proteins such as CRRY or CR2-fH have shown robust efficacy in preclinical IRD models. In a Stargardt disease mouse model, subretinal delivery of AAV-CRRY increased local complement regulation, significantly reduced C3/C3b deposition, slowed photoreceptor degeneration, and improved visual chromophore levels, directly linking complement attack to disease progression and demonstrating structural and functional rescue [80]. Similarly, AAV-mediated delivery of CR2-fH to the RPE reduced excessive complement activation, improved RPE and Bruch’s membrane integrity, and preserved visual function in models of RPE damage [92]. Similar strategies are being explored for other IRDs and AMD, with the goal of achieving long-term complement control from a single treatment [93,94].

Overall, both pharmacologic and gene therapy-based complement modulators have shown the ability to reduce inflammation, preserve retinal structure, and delay secondary degeneration in preclinical IRD models. While human efficacy data in IRDs are still emerging, the mechanistic rationale, promising preclinical results, and favorable safety profiles in related retinal diseases support complement modulation as a promising adjunctive or combination therapy for IRDs [87,89].

## 4. Combination Therapeutic Strategies

**Figure 3:**
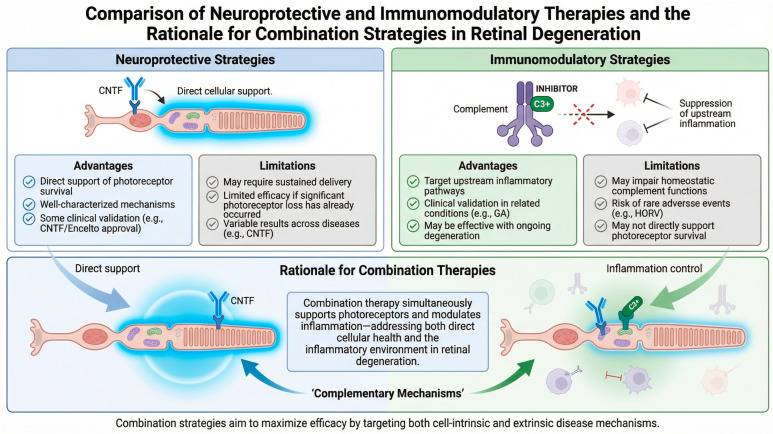
Schematic comparing the advantages and limitations of neuroprotective and immunomodulatory therapeutic pathways for IRD.

The relative advantages and limitations of neuroprotective versus immunomodulatory approaches are useful to compare before discussing combination strategies (Figure 3). These complementary profiles provide the rationale for combination approaches that simultaneously address both direct cellular support and the inflammatory environment.

### 4.1. Rationale for Multi-Target Approaches

The majority of IRDs are characterized by complex, multifactorial pathophysiology involving interconnected pathogenic pathways, including oxidative stress, mitochondrial dysfunction, inflammation, excitotoxicity, and multiple forms of cell death. This complexity provides a strong rationale for multi-target therapeutic approaches that address complementary mechanisms simultaneously, potentially achieving synergistic neuroprotection that exceeds the efficacy of single-agent therapies.

The convergence of multiple pathogenic pathways in IRDs is well established. Photoreceptor cell death in IRDs is driven by calcium dyshomeostasis and excitotoxicity, oxidative stress and mitochondrial dysfunction, and neuroinflammation, mechanisms that are common to all neurodegenerative diseases [95,96,97]. These pathways are not independent but rather interact in positive feedback loops: for example, oxidative stress activates inflammatory responses, which in turn exacerbate mitochondrial dysfunction and cell death [98]. Similarly, photoreceptor degeneration triggers microglial activation and the release of pro-inflammatory cytokines such as TNFα, which further promotes cell death through multiple mechanisms [99].

Multi-target approaches offer the potential for additive effects by simultaneously interrupting multiple nodes in these interconnected pathways. The concept of combination therapy has been highly successful in treating chronic diseases such as cancer, AIDS, hypertension, glaucoma, and Parkinson’s disease, where targeting complementary mechanisms produces superior outcomes compared to monotherapy [95]. In IRDs, preclinical studies have demonstrated that combined inhibition of three enzymes that act sequentially in photoreceptor degeneration can reduce cell death more effectively than single-agent treatment [100]. Similarly, modulation of multiple innate immune pathways has emerged as a promising strategy to prevent or delay vision loss in retinal degenerative diseases [93]. Furthermore, combinations of antioxidants can slow rod photoreceptor degeneration in *rd1* mice more effectively than single agents, and simultaneous inhibition of both the Fas and autophagy pathways produces greater protective effects in models of autosomal dominant RP (adRP) [101,102].

Gene-agnostic, multi-target therapies are particularly attractive for IRDs given the extreme genetic heterogeneity of these disorders. With over 280 genes implicated in IRDs, developing individualized gene therapies for each mutation is impractical and costly [10]. In contrast, targeting common downstream pathways such as oxidative stress, inflammation, and metabolic failure offers potential clinical benefits to all IRD patients irrespective of their specific genetic defect [103]. This approach is especially valuable for patients with advanced disease or unknown genetic mutations, where gene replacement is not feasible.

Finally, by targeting complementary mechanisms, combination therapies may achieve therapeutic effects at lower doses compared to monotherapies, thereby potentially improving safety profiles by reducing the risk of dose-dependent toxicity while maintaining or enhancing efficacy outcomes [95].

### 4.2. Promising Combinations

Multi-target therapeutic strategies combining neuroprotective, anti-inflammatory, metabolic, and immunomodulatory agents to achieve synergistic effects are under early development to treat IRDs. Combining neurotrophic factors with anti-inflammatory agents addresses two critical pathogenic mechanisms in IRDs. Neurotrophic factors such as CNTF, BDNF, and FGFs promote photoreceptor survival through activation of pro-survival signaling pathways, while anti-inflammatory agents reduce microglial activation and cytokine release [78,98]. For example, CNTF not only activates the JAK/STAT pathway in photoreceptors but also modulates Müller glial responses and reduces neuroinflammation, providing dual neuroprotective and anti-inflammatory effects. Similarly, BDNF has been shown to modulate neuroinflammation and support synaptic integrity in addition to its direct anti-apoptotic effects on photoreceptors.

PEDF is a multifunctional protein with neuroprotective, anti-angiogenic, and anti-inflammatory properties, while anti-complement treatments further target the inflammatory cascade that contributes to photoreceptor loss [104,105]. Combining these approaches could provide potentiated benefit by simultaneously supporting photoreceptor survival and reducing complement-mediated inflammation and MAC formation [45,93]. This strategy is particularly relevant for IRDs such as Stargardt disease and RP, where complement dysregulation has been documented [106].

Combining other neurotrophic growth factors (e.g., CNTF, BDNF, GDNF) with complement pathway regulators addresses both the loss of trophic support and the chronic inflammatory environment in degenerating retinas. Growth factors promote photoreceptor survival and metabolic support, while pathological complement suppression reduces microglial recruitment, cytokine release, and MAC-mediated cell death. This combination may be particularly effective in advanced IRDs, where both photoreceptor loss and inflammation are prominent.

Oxidative stress is another central pathogenic mechanism in IRDs, and combining antioxidants with neuroprotective factors targets both the upstream cause (reactive oxygen species accumulation) and downstream consequences (cell death signaling) of retinal degeneration [14]. In a rabbit model, PEDF has demonstrated efficacy in blunting the damage to photoreceptors caused by paraquat, a known oxidant [107]. In addition to PEDF, N-acetylcysteine (NAC), a potent antioxidant, is currently in clinical trials for RP and has shown promise in reducing oxidative damage and slowing photoreceptor loss. In the phase 1 FIGHT RP trial (NCT03063021), oral NAC proved to be safe and well tolerated in patients with moderately advanced RP, with dose-dependent improvements in visual acuity and macular sensitivity over 24 weeks [108,109]. Based on these promising phase 1 results, NAC Attack, a phase 3 randomized, placebo-controlled trial (NCT05537220) is currently active but not recruiting. Evidence for oxidative stress being a key component of photoreceptor cell death in IRDs comes from the observations in end stage choroideremia patients. In these patients, the photoreceptors entirely degenerate but so too does the choroid. As a result, the foveal cones can survive for many years, indeed decades, despite advanced outer retinal loss in the periphery. The difference between choroideremia and most other end-stage IRDs is that the choroid also degenerates due to the RPE loss in choroideremia and this would naturally reduce the oxygen levels in the subretinal space [110,111].

Oral Tinlarebant, which is an inhibitor of vitamin A accumulation, has also shown phase 3 success in the DRAGON trial (NCT05244304) for Stargardt disease meeting its primary efficacy endpoint of a 36% reduction in the growth rate of retinal lesions, measured as definitely decreased autofluorescence (DDAF) by fundus autofluorescence imaging, compared to placebo. Combining NAC or Tinlarebant with neurotrophic factors such as PEDF, CNTF, or RdCVF could enhance mitochondrial integrity, reduce oxidative stress, and provide metabolic support to photoreceptors, potentially achieving synergistic neuroprotection [112].

A comprehensive, multi-target strategy using repurposed drugs that simultaneously address multiple pathogenic mechanisms shared across all retinal degenerations represents a rational and promising therapeutic approach [95]. For example, pharmacology-based drug repurposing and combination treatments have shown mutation-agnostic efficacy in retinopathy models, improving photoreceptor survival and function across diverse genetic backgrounds [113]. Other solutions such as combining a calcium channel blocker (to reduce excitotoxicity), an antioxidant (to mitigate oxidative stress), and an anti-inflammatory agent (to suppress microglial activation) could provide broad-spectrum neuroprotection across different IRDs. This strategy has shown promise in preclinical models and is being explored for translation to clinical trials [95].

In summary, multi-target therapeutic approaches for IRDs are grounded in the complex, multifactorial pathophysiology of these diseases and offer the potential for synergistic neuroprotection by simultaneously addressing oxidative stress, inflammation, metabolic failure, and cell death. The analogy would be glaucoma, where individual genetic mechanisms are not targeted, but broader therapies are applied to successfully prevent disease progression. This includes, for instance, increasing outflow through the trabecular meshwork and uveoscleral pathway, as well as reducing aqueous production (carbonic anhydrase inhibitors) or vitreous volume (osmotic agents such as mannitol). A similar multifactorial approach is likely to benefit patients who have genetic predisposition to photoreceptor neuronal cell loss. Promising combination strategies include neuroprotection plus anti-inflammation, neurotrophins combined with anti-complement therapy, and antioxidants combined with growth factors, all of which are supported by robust preclinical evidence and are advancing toward clinical translation.

## 5. Conclusions and Future Directions

Gene-agnostic approaches offer broad therapeutic potential for IRDs due to the extreme genetic and phenotypic heterogeneity of these disorders. With over 280 causative genes and thousands of unique variants, gene-specific therapies cannot feasibly address the majority of IRD patients. Gene-agnostic strategies, such as neuroprotection, immune modulation, and metabolic support, target common downstream pathways or provide functional rescue independent of the underlying mutation. Gene-agnostic strategies can potentially benefit patients across a range of genotypes and disease stages by targeting common downstream pathogenic mechanisms [9,10,114]. Patients in the early stage of the disease with adequate photoreceptor reserve are likely to be the optimal candidates for these interventions. These gene-agnostic strategies offer genuine hope to the majority of patients with IRDs who currently have no approved treatment options; however, future optimization of delivery routes, vectors and sustained release systems will be essential to realize therapeutic potential in human IRDs.

However, it is essential to recognize that gene-agnostic approaches do not correct the underlying genetic defect: while offering significant therapeutic potential, they are disease-modifying rather than curative. Because they do not correct the underlying genetic defect, the primary pathogenic process continues, and long-term disease progression may occur despite short-term preservation of retinal structure and function. These interventions are therefore best conceptualized as strategies to slow the rate of degeneration, extend the window of useful vision, and potentially enhance the efficacy of complementary treatments such as gene replacement therapy. Their efficacy is expected to be greatest when viable photoreceptors remain and before extensive retinal remodeling has occurred. The duration of benefit and need for repeated treatment remain important questions for ongoing and future clinical trials.

Combination strategies may provide superior outcomes by simultaneously addressing multiple pathogenic mechanisms that converge in IRDs. Recent preclinical and translational studies demonstrate that multi-target therapies, including combinations of neuroprotective agents, anti-inflammatory drugs, and metabolic modulators, can achieve synergistic effects and broader efficacy than monotherapies. Combining neuroprotective and immunomodulatory mechanisms within a single combination therapeutic approach represents a compelling strategy, as it could address two distinct but converging pathogenic pathways with a single intervention, potentially achieving synergistic benefit while simplifying clinical delivery. This paradigm is supported by the success of combination therapies in other chronic diseases and is increasingly reflected in IRD clinical trial pipelines. While combination approaches offer potential for synergistic benefit, they also carry the risk of additive or unpredictable adverse effects. The interaction between neuroprotective and immunomodulatory agents requires careful evaluation, as excessive immunosuppression combined with growth factor delivery could theoretically promote unwanted cell proliferation or mask early signs of inflammation. Systematic dose-finding studies and careful safety monitoring will be essential as combination strategies advance to clinical development.

Important challenges remain, including establishing optimal dosing regimens, demonstrating long-term durability of therapeutic effect, and identifying which patients and disease stages are most likely to benefit. For instance, specific IRDs may respond to complement inhibition or neurotrophins better than others, and late-stage disease may be refractory to neuroprotective and immunomodulatory interventions due to the fundamental requirement for viable target cells. Gene-agnostic therapies that support photoreceptor survival or dampen inflammation cannot restore cells that have already been lost. Furthermore, advanced disease is often accompanied by extensive retinal remodeling, including synaptic rewiring, neuronal migration, and glial scarring, which may disrupt the cellular architecture necessary for functional benefit even if remaining cells are preserved. These considerations underscore that appropriate patient selection and disease staging are critical for therapeutic success, and that earlier intervention is likely to yield better outcomes. Rigorous clinical trial design and appropriate endpoint selection will also be essential to address these questions. Fortunately, from a regulatory standpoint, a single vector expressing multiple transgenes does not trigger the increased burden that the FDA imposes on combination drug therapies [115]. However, it remains unclear whether the FDA will allow an anatomic primary endpoint for IRD approval, as it has for MacTel and potentially for GA [116]. European agencies tend to only approve functional endpoints. Given the potential for a disconnect between retinal cell preservation and visual function, as for instance seen with the revakinagene taroretcel-lwey trials, a functional outcome may be required [59].

There is a critical need for continued investment in biomarker development and clinical trial infrastructure to accelerate the translation of these therapies. Validated anatomical, physiological, and functional biomarkers are essential for reliably measuring treatment effects, stratifying patients, and optimizing trial design. Emerging endpoints with advanced imaging, adaptive optics, and novel functional assays are being incorporated into IRD trials to better capture clinically meaningful changes [117,118]. Robust clinical trial networks and standardized assessment protocols will be vital for efficiently evaluating gene-agnostic and combination therapies across diverse patient populations. Initiatives such as the NCATS-led Platform Vector Gene Therapy (PaVe-GT) pilot project, which aims to streamline and standardize AAV manufacturing methods, may help reduce development costs and accelerate the path to clinical translation for both gene-agnostic and gene-specific therapies targeting rare disease populations [119].

Looking ahead, combination therapies that integrate neuroprotection with anti-inflammatory and metabolic support may yield superior functional outcomes and longer-lasting vision preservation for many IRD patients. While these approaches bypass the need for mutation-specific interventions, responsiveness may still vary depending on the underlying disease mechanism. For example, conditions with primary metabolic defects may respond differently to neuroprotective strategies than those with structural or developmental abnormalities. Future research should aim to identify biomarkers that predict treatment response and guide patient selection. By bypassing the need for individualized gene correction, gene-agnostic and combination strategies can be deployed more broadly, rapidly and affordably, addressing the unmet needs of patients who lack access to gene-specific therapies, whether due to unidentified mutations, rare genotypes without active development programs, or presentation at advanced disease stages. As the therapeutic landscape evolves, these modalities are poised to transform IRD care, offering hope for vision preservation and restoration to a much wider spectrum of patients.

## Figures and Tables

**Table 1:** Gene-Agnostic Therapeutic Approaches for Inherited Retinal Diseases.

Therapeutic Factor	Category	Key Mechanism of Action	Development Status	Key Clinical Trials
PEDF	Neuroprotection	Neurotrophic, anti-angiogenic, and anti-inflammatory properties; protects photoreceptors via suppression of apoptotic pathways	Preclinical	N/A
CNTF	Neuroprotection	Activates neuroprotective signaling via STAT3 pathway; promotes photoreceptor survival	FDA Approved (Encelto for MacTel Type 2)	MacTel: Phase 3 NTMT-03-A (NCT03316300), NTMT-03-B (NCT03319849) RP: Phase 2 NCT01530659, Phase 2/3 NCT00447980, Phase 2 NCT00447993 Glaucoma: Phase 1 NCT01408472, Phase 2 NCT04577300, Phase 2 NCT02862938 GA: Phase 2 NCT00447980 Achromatopsia: Phase 1/2 NCT01648452
RdCVF	Neuroprotection	Rod-secreted factor promoting cone survival; promotes glucose uptake	Clinical trials	RCD: Phase 1/2 PRODYGY (NCT05748873)
BDNF	Neuroprotection	Promotes neuronal survival via TrkB receptor signaling; supports photoreceptor viability	Preclinical	N/A
FGF	Neuroprotection	Multiple growth factors supporting retinal neuron survival and development	Preclinical	N/A
GDNF	Neuroprotection	Glial-derived factor promoting photoreceptor survival	Preclinical	N/A
Proinsulin	Neuroprotection	Activates survival pathways; reduces oxidative stress in photoreceptors	Preclinical	N/A
Complement C3 inhibitors	Immunomodulation	Blocks complement cascade at C3 level; reduces inflammation and cell damage	FDA Approved (Syfovre for GA)	GA: Phase 3 OAKS (NCT03525613), DERBY (NCT03525600)
Complement C5 inhibitors	Immunomodulation	Inhibits terminal complement pathway; prevents membrane attack complex formation	FDA Approved (Izervay for GA)	GA: Phase 3 GATHER1 (NCT02686658), GATHER2 (NCT04435366) Autosomal recessive STGD1: Phase 2b (NCT03364153)
Soluble complement regulators	Immunomodulation	Gene therapy delivery of endogenous complement regulatory proteins	Preclinical/Early clinical	N/A

Abbreviations: RP, retinitis pigmentosa; STGD1, Stargardt disease 1; RCD, rod-cone dystrophy; PEDF, pigment epithelium-derived factor; CNTF, ciliary neurotrophic factor; RdCVF, rod-derived cone viability factor; BDNF, brain-derived neurotrophic factor; FGF, fibroblast growth factor; GDNF, glial cell line-derived neurotrophic factor; GA, geographic atrophy; MacTel, macular telangiectasia; N/A, not applicable.

## Data Availability

No new data were created or analyzed in this study. Data sharing is not applicable to this article.
